# Validation of an IFNγ/IL2 FluoroSpot assay for clinical trial monitoring

**DOI:** 10.1186/s12967-016-0932-7

**Published:** 2016-06-14

**Authors:** Nina Körber, Uta Behrends, Alexander Hapfelmeier, Ulrike Protzer, Tanja Bauer

**Affiliations:** Institute of Virology, Technische Universität München/Helmholtz Zentrum München, Schneckenburgerstr. 8, 81675 Munich, Germany; Clinical Cooperation Group, Immune Monitoring, Helmholtz Zentrum München/Technische Universität München, Munich, Germany; Clinical Cooperation Group Pediatric Tumor Immunology, Children‘s Hospital, Technische Universität München/Helmholtz Zentrum München, Munich, Germany; German Center for Infection Research (DZIF), Partner Site Munich, Munich, Germany; Institute of Medical Statistics and Epidemiology, Technische Universität München, Munich, Germany

**Keywords:** Assay precision, Assay validation, Clinical trial monitoring, EBV-specific T-cell responses, FluoroSpot

## Abstract

**Background:**

The FluoroSpot assay, an advancement of the ELISpot assay, enables simultaneous measurement of different analytes secreted at a single-cell level. This allows parallel detection of several cytokines secreted by immune cells upon antigen recognition. Easier standardization, higher sensitivity and reduced labour intensity render FluoroSpot assays an interesting alternative to flow-cytometry based assays for analysis of clinical samples. While the use of immunoassays to study immunological primary and secondary endpoints becomes increasingly attractive, assays used require pre-trial validation. Here we describe the assay validation (precision, specificity and linearity) of a FluoroSpot immunological endpoint assay detecting Interferon γ (IFNγ) and Interleukin 2 (IL2) for use in clinical trial immune monitoring.

**Methods:**

We validated an IFNγ/IL2 FluoroSpot assay to determine Epstein-Barr virus (EBV)-specific cellular immune responses (IFNγ, IL2 and double positive IFNγ + IL2 responses), using overlapping peptide pools corresponding to EBV-proteins BZLF1 and EBNA3A. Assay validation was performed using cryopreserved PBMC of 16 EBV-seropositive and 6 EBV-seronegative donors. Precision was assessed by (i) testing 16 donors using three replicates per assay (intra-assay precision/repeatability) (ii) using two plates in parallel (intermediate precision/plate-to-plate variability) and (iii) by performing the assays on three different days (inter-assay precision/reproducibility). In addition, we determined specificity, linearity and quantification limits of the assay. Further we tested precision across the two assay systems, IFNγ/IL2 FluoroSpot and the corresponding enzymatic single cytokine ELISpot.

**Results:**

The validation revealed: (1) a high intra-assay precision (coefficient of variation (CV) 9.96, 8.85 and 13.05 %), intermediate precision (CV 6.48, 10.20 and 12.97 %) and reproducibility (CV 20.81 %, 12,75 % and 12.07 %) depending on the analyte and antigen used; (2) a specificity of 100 %; (3) a linearity with *R*^*2*^ values from 0.93 to 0.99 depending on the analyte. The testing of the precision across the two assay systems, adduced a concordance correlation coefficient *p*_*c*_ = 0.99 for IFNγ responses and *p*_*c*_ = 0.93 for IL2 responses, indicating a large agreement between both assay methods.

**Conclusions:**

The validated primary endpoint assay, an EBV peptide pool specific IFNγ/IL2 FluoroSpot assay was found to be suitable for the detection of EBV-specific immune responses subject to the requirement of standardized assay procedure and data analysis.

**Electronic supplementary material:**

The online version of this article (doi:10.1186/s12967-016-0932-7) contains supplementary material, which is available to authorized users.

## Background

The enzyme-linked immuno spot (ELISpot) assay, which enumerates peripheral blood mononuclear cells releasing cytokines upon specific antigen stimulation, has become an assay of choice for evaluation of cell-mediated immune responses in many clinical trials [1–3].

The ELISpot assay is limited, however, in that only one cytokine at a time can be assessed. The FluoroSpot assay, an advancement of the ELISpot assay, enables simultaneous measurement of different analytes secreted at a single-cell level [4, 5]. This facilitates the detection of cells secreting several cytokines in parallel such as e.g. polyfunctional T cells, which have been suggested to be correlates of protection in various infectious diseases [6–8]. By detecting different cytokines with a specific fluorophore and analyzing differentially fluorescent spots by specific filter systems, cells producing single or multiple cytokines can be identified. FluoroSpot assays maintain the simplicity and sensitivity of the ELISpot assay but offer the advantage of multiplex analyses.

Investigating antigen specific immune responses as a primary endpoint in clinical trials requires highly sensitive and validated assays to determine immune cell reactivity ex vivo correlating with clinical outcome. Assays for detecting cellular immune responses in humans have already been used to determine primary endpoints in clinical trials [9, 10], but the validation of these assays has often not been approached in a manner that follows the assay validation guidelines provided for industry [11] (http://www.fda.gov/downloads/drugs/guidancecomplianceregulatoryinformation/guidances/ucm368107.pdf).

Validation is a well-known process in industry, but is much less common for immune monitoring assays used in the academic and clinical settings with only few published guidelines, especially for validation of assays that are considered to be “state-of-art” [12–15]. Since 2005, two consortia have performed proficiency panel experiments to address T cell immunoassay harmonization and as a consequence the MIATA (“Minimum Information About T-cell Assays”) initiative was launched to optimize assay performance and reproducibility between different laboratories [16–18]. Implementation of cross-laboratory validation is known to support reduction of data variability, thus guaranteeing consistency of datasets generated by different clinical trials sites [19–21].

Guidelines for assay validation define eight parameters that must be investigated in order to validate a bioanalytical assay [22]: (1) specificity, (2) accuracy, (3) precision (repeatability, intermediate precision, reproducibility), (4) detection limit, (5) quantification limit, (6) linearity, (7) range and (8) robustness. Here we provide, to our knowledge, the first validation report for an IFNγ/IL2 FluoroSpot assay designed to allow qualitative and quantitative evaluation of cellular immune responses. Further we tested precision across the two related assay systems, IFNγ/IL2 FluoroSpot and the corresponding enzymatic single cytokine ELISpot.

As a result, the validated primary endpoint assay, an Epstein-Barr virus (EBV) peptide pool specific IFNγ/IL2 FluoroSpot assay was found to be suitable for the detection of EBV-specific immune responses in a clinical trial setting.

## Methods

The authors acknowledge the concept of the MIATA framework which was recently published [17, 18]. Therefore, detailed information is provided as structured in the five modules proposed by MIATA (http://www.miataproject.org/) [18].

### The sample

#### Subjects

Peripheral blood was taken by venipuncture from 30 healthy donors (20 women, 10 men) with an average age of 30 years (range 17–50 years). Donors were either EBV-seropositive or EBV-seronegative, diagnosed by pre-testing with a diagnostic assay. A pre-screening of donors for EBV-specific cell-mediated immune responses was done by ELISpot analyses prior to the FluoroSpot validation experiments, to ensure an inclusion of a broad range of EBV-specific low- and high-responders. Informed consent was obtained from all participating subjects prior to their inclusion in this validation experiments.

#### Cryoconservation of PBMC

Within 4 h after collection of heparinized whole blood human peripheral blood mononuclear cells (PBMC) were separated by Ficoll density gradient (human Pancoll, PAN-BIOTECH, Aidenbach, Germany) using 50 ml Leucosep^TM^ tubes (Greiner Bio-One, Frickenhausen) and washed one time with sterile phosphate buffered saline (PBS) (Life Technologies, Darmstadt, Germany) and once with RPMI1640 medium (Life Technologies, Invitrogen, Darmstadt, Germany) following our established standard operating procedure (SOP). Trypan blue (Life Technologies, Darmstadt, Germany) staining was used to count on living cells. The median PBMC number obtained per ml whole blood was 0.7x10^6^ PBMC. PBMC were frozen at 5 × 10^6^ PBMC per vial in 1.8 ml cryotubes (Thermo Scientific, Roskilde, Denmark) in a concentration of 1 × 10^7^ PBMC per 1 ml freezing medium (fetal calf serum (FCS) (Life Technologies, Darmstadt, Germany) supplemented with 10 % dimethyl sulfoxide (Sigma-Aldrich, Steinheim, Germany) using a freezing container (Mr. Frosty, Thermo Scientific, Roskilde, Denmark) and put on −80 °C. After 24 h PBMC were stored in liquid nitrogen until further use.

#### Thawing and resting of PBMC

According to our SOP, PBMC were thawed at 37 °C using Roswell Park Memorial Institute (RPMI1640) medium supplemented with 10 % FCS and 1 % penicillin–streptomycin (PenStrep, Life Technologies, Invitrogen, Darmstadt, Germany) (abbr.: RPMI-10). After two washing steps with RPMI-10, cells were counted with an automated cell counter (Vi-cell XR, Beckman Coulter, Krefeld, Germany). The median cell recovery after thawing was 5.0 × 10^6^ PBMC per vial with a median viability of 93 %. For a standard resting procedure PBMC were incubated for 18 h at 37 °C in a humidified atmosphere at 5 % CO_2_ at a concentration of 2 × 10^6^ PBMC/ml RPMI-10. After resting the median cell recovery was 4.8 × 10^6^ PBMC per vial with a median viability of 95 %.

### The assay

#### Stimulatory agents

The following stimulatory agents were used in this study: Overlapping peptide pools of EBV-derived proteins BZLF1 (59 peptides) and EBNA3A (234 peptides) (JPT Peptide Technologies, Berlin, Germany), consisting of 15mers overlapping 11 amino acids in a concentration of 1 µg/ml. The optimal assay concentration of both peptide pools was identified in previous titration experiments. Phytohemagglutinin (PHA-L) (Sigma-Aldrich Chemie, Schnelldorf, Germany) was used as a mitogen for stimulation in a concentration of 2 µg/ml. All experiments were performed in triplicates when cells were stimulated with antigen (BZLF1, EBNA3A) or in six replicates for the PHA-L-stimulated cells. RPMI-10 was added as a negative control in triplicates and anti-CD3 (in a dilution of 1:1000, mAb CD3-2, Mabtech AB, Nacka Strand, Sweden) was used as a positive control in a single well for each donor.

#### IFNγ/IL2 FluoroSpot assay

IFNγ/IL2 FluoroSpot assays (human IFNγ/IL2 FluoroSpot Kit with pre-coated plates, product code: FSP-0102-10, Mabtech AB, Nacka Strand, Sweden) were performed according to the manufacturer´s instructions, except for washing steps, which were increased to a seven-time washing. Either 2 × 10^5^ PBMC/well for BZLF1- and EBNA3A-stimulated PBMC or 5 × 10^4^ PBMC/well for PHA-L-stimulated PBMC were plated in a final volume of 150 µl/well.

#### ELISpot assays

IFNγ ELISpot assays (human IFNγ ELISpot^PLUS^ Kit with pre-coated plates, product code: 3420-4APW-10, Mabtech AB, Nacka Strand, Sweden) and IL2 ELISpot assays (human IL2 ELISpot^PLUS^ Kit with pre-coated plates, product code: 3445-4APW-10, Mabtech AB, Nacka Strand, Sweden) were performed according to the manufacturer´s instructions, except for washing steps, which were increased to a seven-time washing. 2 × 10^5^ PBMC/well were plated in a final volume of 150 µl/well and stimulated with peptide pools of EBV-derived proteins BZLF1 or EBNA3A, respectively.

#### Data acquisition

**Fig. 1:**
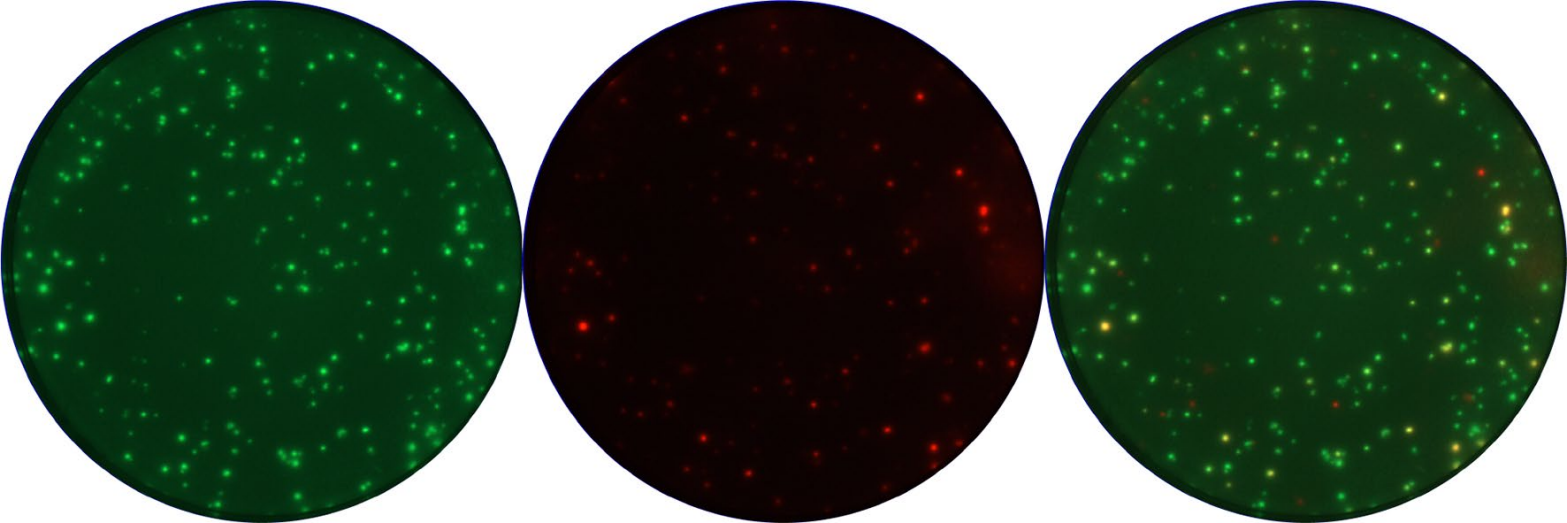
Illustration of IFNγ/IL2 FluoroSpot assay images. Individual images were captured for IFNγ (*left image*) (FITC filter) and IL2 (*central image*) (PE filter) and used to generate the computerized overlay of the two filters showing double positive IFNγ + IL2 cell responses (*right image*). IFNγ, IL2 and IFNγ + IL2 secreting cells upon stimulation with BZLF1 are depicted as *green* (*left image*), *red* (*central image*), and *yellow* (*right image*) spots, respectively

ELISpot and FluoroSpot plates were evaluated within 3 days after assay performance using an automated reader system (CTL-ImmunoSpot^®^ S6 Ultra-V Analyzer/CTL ImmunoSpot 5.1 Professional DC Software, CTL Europe, Bonn, Germany). ELISpot plates were scanned with automatically adjusted settings conducted by the reader. Solely the selection of the plate type and the centring of the wells were done manually. FluoroSpot plates were scanned with manual settings for both fluorophore filters (fluorescein isothiocyanate (FITC)/phycoerythrin (PE)) by adjusting the gain and exposure time of the UV-light. Counting of spot forming cells (SFC) within ELISpot and FluoroSpot plates was performed manually in compliance with the guidelines for the automated evaluation of ELISpot assays [23] and our laboratory standard counting parameters consisting of a best possible spot separation, a spot size gating from minimum to maximum and a counting mask size of 90 %. Spot counts were normalized to 100 % of the well area. The settings for sensitivity of spot counting were established and adjusted manually for each plate using antigen stimulated and negative control wells. In detail, the sensitivity for the counting of single spots was adjusted by identifying antigen-stimulated wells with spots that were well distributed and clearly distinguishable from background activity and artefacts. In a next step, the selected parameters for the sensitivity of counting spots were checked on negative control wells to prevent to count on small background spots. If necessary, the parameters for the sensitivity of counting spots were adjusted to exclude artefact or background spots. The performed adjustments were rechecked on the antigen-stimulated wells and if necessary adapted another time to make sure to count on the most distinct spots. This way of parameter checking was repeated on other sets of antigen-stimulated and negative control wells. If possible, throughout the counting procedure of one single plate, similar settings were used for replicates of one donor, and one antigen. All obtained counts were reviewed and certified by a second person during a quality control process including an exclusion of artefacts within wells or a rejection of failed wells. Figure 1 displays an image example of representative data sets obtained using the IFNγ/IL2 FluoroSpot assay in which cryopreserved PBMC of an EBV-seropositive donor were stimulated with the BZLF1 peptide pool.

### Interpretation of results

Final results are represented as spot forming cells (SFC) per 2 × 10^5^ PBMC for BZLF1- and EBNA3A-stimulated PBMC and 5 × 10^4^ PBMC for PHA-L-stimulated PBMC. Unless specified differently, denoted results represent background subtracted data. The median background reactivity (spot counts in negative control wells) observed within the ELISpot assay was 0 spots per well (range 0–4 SFC/well) in IFNγ ELISpot assays and 5 spots per well (range 0–19 SFC/well) in IL2 ELISpot assays. For the IFNγ/IL2 FluoroSpot assays we observed a median background of 1 spot per well (range 0–4 SFC/well) for single positive IFNγ responses (IFNγ), 5 spots per well (range 1–10 SFC/well) for single positive IL2 responses (IL2) and 1 spots per well (range 0–2 SFC/well) for double positive IFNγ + IL2 responses (IFNγ + IL2). Positive reactivity to experimental stimulatory agents was selected as a *p*-value of equal or smaller than 0.05 when applying the distribution free sampling method (DFR(2x)), by using a web-based tool (http://www.scharp.org/zoe/runDFR/) [24], which compares the spot counts in antigen stimulated wells with spot counts in negative control wells. In addition, only mean spot counts of at least 11 SFC/2 × 10^5^ PBMC (in EBV peptide pool stimulated wells) or 11 SFC/5 × 10^4^ PBMC (in PHA-L-stimulated wells) were regarded as a positive reactivity. Outliers of the replicates, predefined as results which originate from irregular wells, were excluded during quality control. Raw data of all performed assays can be provided upon request.

### Laboratory environment

All experiments were performed by well-trained members of the lab in accordance with our established SOP protocols. Laboratory personnel participated regularly in external international ELISpot proficiency panels.

### Statistical analyses

To define assay precision, the coefficient of variation (%CV) was calculated as the ratio of the standard deviation to the mean and expressed as a percentage value. All tests were two-sided and were conducted on exploratory 5 % significance levels. Effect measures are presented with 95 % confidence intervals. Linear regression analysis was performed to assess the coefficient of determination *R*^*2*^. The concordance correlation coefficient *p*_*c*_ by Lin [25] was calculated to investigate agreement of measurements. Likewise, the Bland–Altman method was used to assess the agreement between FluoroSpot and ELISpot measurements by calculating the average difference *d* (FluoroSpot-ELISpot) and the 95 % limits of agreement (*d* ± 1.96 standard deviation (*s*) of the difference) [26]. The software Graph Pad Prism 5.00 (GraphPad Software, La Jolla, California, USA) and R (http://www.r-project.org/) [27] were used for statistical analyses.

## Results

First we evaluated assay precision, including repeatability (intra-assay precision), intermediate precision (plate-to-plate variability) and reproducibility of the assay (day to day variability). For the complete validation process cryopreserved PBMC of one isolation batch were used to assess assay precision using the same lot of assay reagents. All samples were assayed in triplicates (BZLF1 and EBNA3A) or six replicates (PHA-L; control antigen).

### Intra-assay precision

We used PBMC of 16 donors to examine intra-assay precision of the IFNγ/IL2 FluoroSpot assay. Overall we determined a high intra-assay precision for IFNγ, IL2 and IFNγ + IL2 responses for both, the EBV-derived antigens and the mitogen. Regarding all donors, a mean CV of 9.96, 8.85 and 13.05 % was obtained for IFNγ, IL2 and IFNγ + IL2 responses, respectively (Table 1A–C). These results indicate that intra-assay variability was acceptable. It also justified the analy

**Table 1:** Intra-assay variability of IFNγ, IL2 and IFNγ + IL2 responses in the IFNγ/IL2 FluoroSpot assay

Donor	Antigen	Replicate 1	Replicate 2	Replicate 3	Replicate 4	Replicate 5	Replicate 6	Mean	SD	%CV
A										
S09	BZLF1	283	267	280	ND	ND	ND	277	8.50	3.07
S11	BZLF1	432	440	493	ND	ND	ND	455	33.15	7.29
S14	BZLF1	438	452	479	ND	ND	ND	456	20.84	4.57
S16	BZLF1	458	374	384	ND	ND	ND	405	45.88	11.32
S18	BZLF1	110	148	103	ND	ND	ND	120	24.21	20.12
S01	EBNA3A	30	26	31	ND	ND	ND	29	2.65	9.12
S10	EBNA3A	28	31	31	ND	ND	ND	30	1.73	5.77
S12	EBNA3A	38	43	46	ND	ND	ND	42	4.04	9.55
S20	EBNA3A	68	60	81	ND	ND	ND	70	10.60	15.21
S21	EBNA3A	166	158	192	ND	ND	ND	172	17.78	10.34
S12	PHA-L	35	35	45	44	Rejected	38	39	4.83	12.25
S15	PHA-L	718	659	774	685	679	Rejected	703	45.01	6.40
S21	PHA-L	Rejected	715	836	740	786	771	770	46.20	6.00
S25	PHA-L	293	326	391	250	276	Rejected	307	54.37	17.70
S26	PHA-L	98	120	123	Rejected	98	123	112	13.20	11.75
S27	PHA-L	253	280	284	264	226	Rejected	261	23.38	8.95
*Mean*									22.27	*9.96*
B										
S09	BZLF1	63	67	52	ND	ND	ND	61	7.77	12.80
S11	BZLF1	28	27	30	ND	ND	ND	28	1.53	5.39
S14	BZLF1	37	31	34	ND	ND	ND	34	3.00	8.82
S16	BZLF1	39	39	43	ND	ND	ND	40	2.31	5.73
S18	BZLF1	32	37	37	ND	ND	ND	35	2.89	8.17
S01	EBNA3A	33	29	21	ND	ND	ND	28	6.11	22.08
S10	EBNA3A	26	26	20	ND	ND	ND	24	3.46	14.43
S12	EBNA3A	47	44	51	ND	ND	ND	47	3.51	7.42
S20	EBNA3A	41	32	Rejected	ND	ND	ND	37	6.36	17.44
S21	EBNA3A	42	38	38	ND	ND	ND	39	2.31	5.87
S12	PHA-L	142	148	143	142	112	Rejected	137	14.42	10.49
S15	PHA-L	718	645	647	718	652	Rejected	676	38.43	5.68
S21	PHA-L	Rejected	685	688	693	725	700	698	16.02	2.29
S25	PHA-L	413	410	408	382	Rejected	412	405	13.00	3.21
S26	PHA-L	488	505	511	554	501	524	514	22.96	4.47
S27	PHA-L	374	423	430	445	385	383	407	29.59	7.28
*Mean*									10.85	*8.85*
C										
S09	BZLF1	50	51	57	ND	ND	ND	53	3.79	7.19
S11	BZLF1	19	18	21	ND	ND	ND	19	1.53	7.90
S14	BZLF1	24	18	18	ND	ND	ND	20	3.46	17.32
S16	BZLF1	26	23	26	ND	ND	ND	25	1.73	6.93
S18	BZLF1	23	29	29	ND	ND	ND	27	3.46	12.83
S01	EBNA3A	18	11	11	ND	ND	ND	13	4.04	30.31
S10	EBNA3A	13	18	11	ND	ND	ND	14	3.61	25.75
S12	EBNA3A	23	25	27	ND	ND	ND	25	2.00	8.00
S20	EBNA3A	10	14	16	ND	ND	ND	13	3.06	22.91
S21	EBNA3A	22	17	21	ND	ND	ND	20	2.65	13.23
S12	PHA-L	0	0	0	0	0	0	0	–	–
S15	PHA-L	Rejected	317	372	345	363	Rejected	349	24.25	6.94
S21	PHA-L	Rejected	460	454	437	Rejected	489	460	21.65	4.71
S25	PHA-L	113	118	124	Rejected	125	Rejected	120	5.60	4.66
S26	PHA-L	66	79	89	68	70	93	78	11.43	14.75
S27	PHA-L	175	181	200	189	142	Rejected	177	21.89	12.34
*Mean*									7.61	*13.05*

Values represent the number of detected antigen-specific IFNγ (A), IL2 (B) and IFNγ + IL2 (C) SFC/2 × 10^5^ PBMC (stimulated with 1 µg/ml BZLF1 or EBNA3A peptide pools) or mitogen-specific IFNγ (A), IL2 (B) and IFNγ + IL2 (C) SFC/5 × 10^4^ PBMC (stimulated with 2 µg/ml PHA-L) in the IFNγ/IL2 FluoroSpot assay (data is not background subtracted); “rejected” wells were not accepted because they did not pass the quality control

*ND* not done, *SD* standard deviation, *CV* coefficient of variation

sis of samples with lower replicates when clinical material is limited.

### Inter-assay precision

To evaluate inter-assay variability we used PBMC of eight donors plated into two different assay plates in parallel. Inter-assay precision of the two assays performed on the same day showed low inter-plate variability. The mean CV for all tested donors was 6

**Table 2:** Inter-assay variability of IFNγ, IL2 and IFNγ + IL2 responses in the IFNγ/IL2 FluoroSpot assay

Donor	Antigen	Plate 1	Plate 2	Plate 1 and plate 2		
		Mean	Mean	Mean	SD	% CV
A						
S09	BZLF1	274	233	254	28.99	11.44
S11	BZLF1	317	326	322	6.36	1.98
S12	EBNA3A	92	94	93	1.41	1.52
S13	EBNA3A	65	63	64	1.41	2.21
S15	PHA-L	645	680	663	24.75	3.74
S21	PHA-L	668	753	711	60.10	8.46
S25	PHA-L	234	287	261	37.48	14.39
S26	PHA-L	214	240	227	18.38	8.10
*Mean*					22.36	*6.48*
B						
S09	BZLF1	56	47	52	6.36	12.36
S11	BZLF1	52	86	69	24.04	34.84
S12	EBNA3A	33	35	34	1.41	4.16
S13	EBNA3A	0	0	0	–	–
S15	PHA-L	575	657	616	57.98	9.41
S21	PHA-L	698	691	695	4.95	0.71
S25	PHA-L	411	403	407	5.66	1.39
S26	PHA-L	547	617	582	49.50	8.50
*Mean*					21.42	*10.20*
C						
S09	BZLF1	52	49	51	2.12	4.20
S11	BZLF1	61	85	73	16.97	23.25
S12	EBNA3A	19	28	24	6.36	27.08
S13	EBNA3A	0	0	0	–	–
S15	PHA-L	329	349	339	14.14	4.17
S21	PHA-L	453	459	456	4.24	0.93
S25	PHA-L	86	105	96	13.44	14.07
S26	PHA-L	102	130	116	19.80	17.07
*Mean*					11.01	*12.97*

Values represent the mean number of antigen-specific IFNγ (A), IL2 (B) and IFNγ + IL2 (C) SFC/2 × 10^5^ PBMC (stimulated with 1 µg/ml BZLF1 or EBNA3A peptide pools) or mitogen-specific IFNγ (A), IL2 (B) and IFNγ + IL2 (C) SFC/2 × 10^5^ PBMC/5 × 10^4^ PBMC (stimulated with 2 µg/ml PHA-L) detected in two different assay plates performed on the same day in parallel

*SD* standard deviation, *CV* coefficient of variation

.48, 10.20 and 12.97 % for IFNγ, IL2 and IFNγ + IL2 responses, respectively (Table 2A–C).

We assessed inter-assay variability also in assays performed on three consecutive days (i.e. reproducibility) using PBMC of ten donors. The mean CV was 20.81, 12.75 and 12.07 % for IFNγ, IL2 and IFNγ + IL2 responses, respectively (Table 3A–C). The inter-assay testing revealed that inter-day variability was only slightly higher than the inter-plate variability for IL2 and IFNγ + IL2 responses. The CV for IFNγ responses, however, was clearly higher when we performed the assay at different days, but still acceptable.

**Table 3:** Inter-day variability of IFNγ, IL2, IFNγ + IL2 responses in the IFNγ/IL2 FluoroSpot assay

Donor	Antigen	Day 1	Day 2	Day 3	Mean	SD	%CV
A							
S09	BZLF1	191	145	149	162	25.48	15.76
S11	BZLF1	326	344	351	340	12.90	3.79
S14	BZLF1	223	161	175	186	32.52	17.45
S18	BZLF1	257	194	189	213	37.90	17.77
S10	EBNA3A	27	27	19	24	4.62	18.98
S12	EBNA3A	92	44	35	57	30.64	53.76
S19	EBNA3A	144	127	67	113	40.45	35.90
S15	PHA-L	304	358	311	324	29.37	9.05
S25	PHA-L	77	88	60	75	14.11	18.81
S26	PHA-L	214	242	297	251	42.23	16.82
*Mean*						27.02	*20.81*
B							
S09	BZLF1	69	62	67	66	3.61	5.46
S11	BZLF1	18	20	17	18	1.53	8.33
S14	BZLF1	48	57	62	56	7.09	12.74
S18	BZLF1	27	29	48	35	11.59	33.43
S10	EBNA3A	17	18	15	17	1.53	9.17
S12	EBNA3A	33	34	29	32	2.65	8.27
S19	EBNA3A	17	12	20	16	4.04	24.74
S15	PHA-L	492	615	572	560	62.42	11.15
S25	PHA-L	344	359	348	350	7.77	2.22
S26	PHA-L	521	617	662	600	72.02	12.00
*Mean*						17.42	*12.75*
C							
S09	BZLF1	69	56	63	63	6.51	10.38
S11	BZLF1	20	19	18	19	1.00	5.26
S14	BZLF1	54	60	54	56	3.46	6.19
S18	BZLF1	32	32	42	35	5.77	16.34
S10	EBNA3A	14	13	13	13	0.58	4.33
S12	EBNA3A	19	26	20	22	3.79	17.47
S19	EBNA3A	12	11	14	12	1.53	12.39
S15	PHA-L	215	276	258	250	31.34	12.55
S25	PHA-L	50	59	47	52	6.24	12.01
S26	PHA-L	95	130	155	127	30.14	23.79
*Mean*						9.04	*12.07*

Values represent the mean number of antigen-specific IFNγ (A), IL2 (B) and IFNγ + IL2 (C) SFC/2 × 10^5^ PBMC (stimulated with 1 µg/ml BZLF1 or EBNA3A peptide pools) or mitogen-specific IFNγ (A), IL2 (B) and IFNγ + IL2 (C) SFC/2 × 10^5^ PBMC/5 × 10^4^ PBMC (stimulated with 2 µg/ml PHA-L) detected on three different assay plates performed on three consecutive days

*SD* standard deviation, *CV* coefficient of variation

Concerning the obtained CVs for low counts of cytokine secreting cells one should keep in mind, that for mathematical reasons, high CV values tend to be determined when spot numbers are of low frequency.

Next, we analyzed the sensitivity of the assay and determined its quantitative range and linearity.

### Limit of detection

The limit of detection (LOD) of the ELISpot/FluoroSpot assay is defined as the lowest number of spots that is precisely distinguishable from an unstimulated control well (*O*) with LOD = *O* + 2SD of *O* [11, 28]. For the IFNγ/IL2 FluoroSpot assay we determined mean background levels of 2 SFC/well (range 0–4, SD 1.37), 5 SFC/well (range 1–10, SD 2.53) and 1 SFC/well (range 0–2, SD 0.54) for IFNγ, IL2, and IFNγ + IL-2 responses, respectively (Additional file 1: Table S1). Based on these results and in line with the determined LLOQ (lower limit of quantification) we set a consistent LOD for the IFNγ/IL2 FluoroSpot assay at 10 SFC/2x10^5^ PBMC.

### Lower limit of quantification

To specify the lower limit of quantification (LLOQ), which is defined as the lowest value that can be quantitatively determined with acceptable precision and accuracy [13], of the IFNγ/IL2 FluoroSpot assay we analyzed the intra-assay CV of antigen-specific IFNγ, IL2 and IFNγ + IL2 responses of donors with numbers of SFC ranging between 1–10 SFC/well. We calculated an intra-assay CV of 46.28, 43.48 and 69.74 % for IFNγ, IL2 and IFNγ + IL2 responses, respectively (Additional file 2: Table S2a–c). These results indicate that SFC counts below 11 SFC/well are of low precision, but it should be taken into account that the high variability in low counts expressed as CV, could be a result of the mathematical equation of the CV, which presents the ratio of the standard deviation to the mean. In contrast, SFC counts of “low responders” ranging between 11–50 SFC/well revealed an acceptable intra-assay CV (<25 %) of 10.70, 9.29 and 16.13 % for IFNγ, IL2, and IFNγ + IL2 responses, respectively (Additional file 3: Table S3a–c). In line with these observations, we set the LLOQ for the IFNγ/IL2 FluoroSpot assay to 11 SFC/well.

### Upper limit of quantification

The automated reader system we used is able to count SFC numbers up to approximately 1000 SFC per well. Intra-assay variability of those high SFC numbers is still acceptable (%CV < 25; data not shown), the setting of counting parameters, however, is difficult, because fusion of several adjacent single spots results in spot aggregates. We used cryopreserved PBMC of six donors to determine the upper limit of quantification (ULOQ), which is defined as the highest value that can be quantitatively determined with acceptable precision and accuracy [11]. All samples were stimulated with PHA-L and assayed in six replicates. Based on the morphology and the ability to count on clearly separated single spots we defined the ULOQ of an IFNγ/IL2 FluoroSpot assay as 700 SFC/well (data not shown). Magnitude of EBV-specific responses was always below this ULOQ.

### Linearity

To determine linearity of the IFNγ/IL2 FluoroSpot assay we tested PBMC of two different donors in six replicates using cell numbers of 1.25 × 10^4^, 2.5 × 10^4^, 5 × 10^4^ and 1 × 10^5^/well. PBMC were stimulated with the mitogen PHA-L, to ensure adequate numbers of IL2-secreting cells. Stimulating PBMC with peptide pools of EBV-derived proteins BZLF1 and EBNA3A did not reveal high enough frequencies of IL2-secreting cells to assess linearity of the IFNγ/IL2 FluoroSpot assay.

**Fig. 2:**
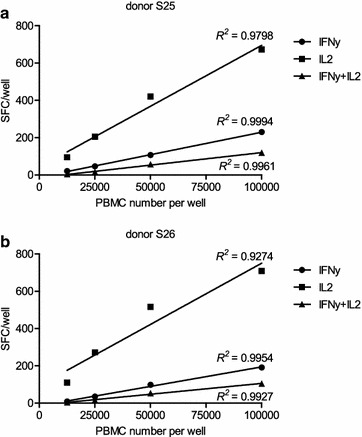
Linearity of the IFNγ/IL2 FluoroSpot assay. Magnitude of IFNγ, IL2, and IFNγ + IL2 responses of donor S25 (**a**) and S26 (**b**) within the IFNγ/IL2 FluoroSpot assay as a function of cell density. Depicted are the number of mitogen-specific IFNγ, IL2 and IFNγ + IL2 SFC/well after stimulation with 2 µg/ml PHA-L; *SFC* spot forming cells. *R* ^*2*^ = coefficient of determination

For both donors we observed a linear relationship for IFNγ (*R*^*2*^ = 0.99, respectively), IL2 (*R*^*2*^ = 0.98 and 0.93, respectively), and IFNγ + IL2 responses (*R*^*2*^ = 0.99, respectively) (Fig. 2). These results suggest that the analysis of less cells per well is feasible, when clinical material is limited. The authors recommend, however, re-assessing linearity using the IFNγ/IL2 FluoroSpot assay for clinical trial monitoring. A lower cell concentration can only be recommended if an appropriate cell-to-cell contact (*e.g.* in a smaller well format (384 well plate)) and an effective way of antigen presentation is ensured, and a true linear correlation between the plated cell number and the spot number exists.

### Diagnostic specificity and sensitivity

Among donors with confirmed positive EBV-serostatus the EBV-specific IFNγ/IL2 FluoroSpot was positive in all 16/16 donors (diagnostic sensitivity: 100 %) with mean frequencies of BZLF1- and EBNA3A-specific T cells ranging from 11–411 SFC/2 × 10^5^ PBMC (median 90 SFC/2 × 10^5^ PBMC) and 12–167 SFC/2 × 10^5^ PBMC (median 54 SFC/2 × 10^5^ PBMC), respectively (Additional file 4: Table S4).

An EBV-specific IFNγ/IL2 FluoroSpot assay with PBMC of the control group (n = 6 EBV-seronegative donors) was negative (Additional file 4: Table S4). This result proved a specificity of the EBV-specific IFNγ/IL2 FluoroSpot assay of 100 %, thus allowing the analysis of EBV-specific immune responses in a clinical trial setting.

### Precision across assays: FluoroSpot vs. ELISpot assay

Finally we tested the concordance between the two assay systems by determining numbers of IFNγ- and IL2-secreting cells in the FluoroSpot- and the corresponding single cytokine ELISpot assay. We used cryopreserved PBMC of ten and eight donors to examine number of IFNγ and IL2 secreting cells, respectively. PBMC stimulated with BZLF1 or EBNA3A were assayed in triplicates.

**Fig. 3:**
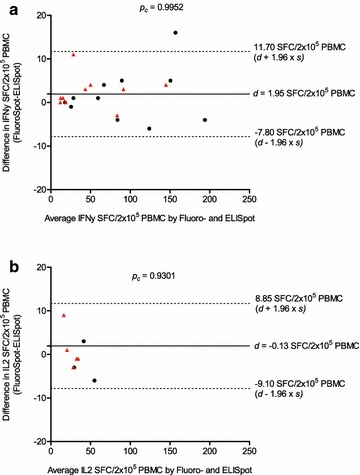
Precision across assays: FluoroSpot vs. ELISpot assay. Concordance between numbers of antigen-specific (BZLF1 and EBNA3A peptide pools) IFNγ- (**a**) and IL2 (**b**) SFC/2x10^5^ PBMC detected within the IFNγ/IL2 FluoroSpot assay and an enzymatic IFNγ and IL2 ELISpot assay. Plotted is the difference in IFNγ (**a**) and IL2 (**b**) SFC/2x10^5^ PBMC detected after ex vivo restimulation with BZLF1 (*black circle*) or EBNA3A (*red triangle*) peptide pools within the FluoroSpot- or ELISpot assay plotted against the average of IFNγ or IL2 SFC detected in either of the two assays. Concordance between FluoroSpot and ELISpot results was assessed using the concordance correlation coefficient *p*_*c*_ by Lin. Descriptive statistics are the average difference *d* (*horizontal solid line*) and the limits of agreement (*d* ± 1.96 × *s*) (*dashed line*) of the detected T cell responses of both assay systems. *d* bias of measurements; *s* standard deviation; *p*_*c*_ concordance correlation coefficient by Lin

Calculating the concordance of the two assay systems we obtained a mean difference *d* between the two assay systems of *d* = 1.95 SFC/2 × 10^5^ PBMC (95 % limits of agreement: −7.80 to 11.70) for IFNγ responses and *d* = −0.13 SFC/2 × 10^5^ PBMC (95 % limits of agreement: −9.10 to 8.85) for IL2 responses with a concordance correlation coefficient *p*_*c*_ = 0.99 and *p*_*c*_ = 0.93, respectively (Fig. 3), indicating that both assay methods give congruent results.

## Discussion

Although assay validation is a time consuming process it is a prerequisite for valid clinical trial monitoring using immunoassays (i.e. ELISpot, FluoroSpot or flow-cytometry-based assays). Following the guidelines for assay validation [22], we determined specificity, precision, detection limit, quantification limit and linearity of an IFNγ/IL2 FluoroSpot assay.

ELISpot assays are very well-suited for high-throughput analyses and have become a standard technique to assess T cell responses within a clinical trial setting [29, 30]. The applicability of the assay system in clinical practice has been confirmed with the approval of a diagnostic ELISpot for the detection of latent tuberculosis infection and disease [31]. Assay performance and data analysis is less time-consuming compared to flow cytometry-based assays (e.g. intracellular cytokine staining). In addition, assay performance can readily be standardized, validated according to international guidelines [11], and quality-controlled in internationally conducted proficiency panels [32, 33] (http://www.proficiencypanel.com/).

A major limitation of the ELISpot assay is its restriction to single parameter analyses. Several studies showed that immune monitoring using single cytokine detection is insufficient to provide an overall assessment of the T cell response and that the identification of protective functional immune signatures requires polyfunctional analysis [34]. Multiparametric FluoroSpot, an advancement of the ELISpot assay, allows the simultaneous assessment of multiple parameters in one well. Currently commercially available FluoroSpot assays are restricted to a maximum of three parameters, but technically up to six parameters are possible.

Some studies have demonstrated that ELISpot assays have a higher CV for intra- and inter-assay precision compared with flow cytometry-based assays (e.g. intracellular cytokine staining, ICS) [35]. In our hands, ELISpot and ICS show a relatively high level of concordance with comparable CV values [36], but we did not test whether this applies also to the IFNγ/IL2 FluoroSpot assay. International proficiency panels have shown that ELISpot assays give reproducible results among different laboratories and the inter-laboratory CV was found to be less than 20 % [37, 38]. For the IFNγ/IL2 FluoroSpot assay we showed intra- and inter-assay precision with CV values clearly within this acceptable level.

Linearity is another important aspect of cell-based immunoassay validation [35]. The IFNγ/IL2 FluoroSpot assay showed a high linearity upon mitogen stimulation of PBMC, offering the possibility of using reduced cell numbers per well. But the minimal applicable number of cells per well must be adjusted for each antigen to the expected frequency of responding cells which may be present at or near the assay detection limit. In addition a check of assay linearity with the respective stimulating-antigen is always required, due to the need for optimal cell-to-cell contact (with e.g. antigen-presenting cells) for sufficient stimulation.

Evaluating the limits of detection and quantification is important when establishing the parameters of an acceptable positive immune response [39]. Theoretically the detection limit of an ELISpot/FluoroSpot assay is extremely low, but due to a high variability at lower concentrations, the detection limit may not be accurate. In contrast, the lowest limit of quantification (LLOQ) is the lowest concentration that can be defined with highest accuracy and precision. It is often postulated that the detection limit of the ELISpot//FluoroSpot assay can be as low as 1/100,000 cells, thus at least ten times lower than ICS [40]. Our results show, however, that CV values are unacceptably high when dealing with these low frequencies of antigen-specific cells. Nonetheless, one should be aware that high variability in low counts expressed by the CV, could be a result of the mathematical equation of the CV, which presents the standard deviation to the mean. For the IFNγ/IL2 FluoroSpot assay we determined a detection limit of 0.005 % which is similar to the LLOQ we determined in previous validation studies for the ELISpot and ICS and what has been reported by others [24].

To address the diagnostic specificity and diagnostic sensitivity of the IFNγ/IL2 FluoroSpot assay for use in an envisaged clinical trial setting we determined these parameters in an existing cohort of individuals with confirmed positive or negative EBV-serostatus. Both specificity and sensitivity was very high, proving the assay very suitable for monitoring in a clinical trial setting as it fulfills the acceptance criteria for biomarker [11] assays.

Finally, we also compared precision across two assay systems, the IFNγ/IL2 FluoroSpot assay and the corresponding enzymatic single cytokine ELISpot assay. We obtained very high concordance between the results of the two assays with an equivalent sensitivity (concordance correlation coefficient *p*_*c*_ = 0.99 and *p*_*c*_ = 0.93 for IFNγ and IL2 responses, respectively) as already reported by others [41]. This allows for comparability of already existing ELISpot-based data (e.g. from previous trial monitoring) with data obtained with the more advanced IFNγ/IL2 FluoroSpot assay.

In summary, the IFNγ/IL2 FluoroSpot assay showed high precision in combination with very high sensitivity and specificity. The broad linear range allows for more flexible specimen volume and permits analysis of fewer cells per assay when clinical material is limited. The IFNγ/IL2 FluoroSpot assay passed all validation checks and is suitable for the detection of EBV-specific immune responses in a clinical trial setting.

## Conclusions

Investigating antigen specific immune responses as a primary endpoint in clinical trials requires highly sensitive and validated assays to determine immune cell reactivity ex vivo correlating with clinical outcome. The FluoroSpot assay enables simultaneous analysis of single cells secreting multiple cytokines thus overcoming an important current limitation of single color enzymatic ELISpot assays. The FluoroSpot assay allows monitoring of polyfunctional T cells, which have been suggested to be correlates of protection in various infectious diseases. Our data show that the validated primary endpoint assay, an IFNγ/IL2 FluoroSpot assay is suitable for the detection of EBV-specific immune responses in a clinical trial setting, subject to the requirement of standardized assay procedure and data analysis.

## Additional files

10.1186/s12967-016-0932-7 Magnitude of background activity measured in unstimulated control wells.
10.1186/s12967-016-0932-7 Intra-assay variability of antigen-specific IFNγ, IL2, and IFNγ + IL2 responses ranging between 1-10 SFC/well.
10.1186/s12967-016-0932-7 Intra-assay variability of antigen-specific IFNγ, IL2, and IFNγ + IL2 responses ranging between 11-50 SFC/well.
10.1186/s12967-016-0932-7 Diagnostic specificity and sensitivity of the EBV-specific IFNγ/IL2 FluoroSpot.

## Abbreviations

CV: coefficient of variation
EBV: Epstein–Barr virus
ELISpot: enzyme-linked immuno spot
FCS: fetal calf serum
FITC: fluorescein isothiocyanate
ICS: intracellular cytokine staining
IFN: interferon
IL: interleukin
LOD: limit of detection
LLOQ: lower limit of quantification
MIATA: minimum information about T-cell assays
PBMC: peripheral blood mononuclear cells
PBS: phosphate buffered saline
PE: phycoerythrin
PHA-L: phytohemagglutinin
RPMI: Roswell Park Memorial Institute
SD: standard deviation
SFC: spot forming cells
SOP: standard operating procedure
ULOQ: upper limit of quantification
