# Supplementary material for: Validation of an IFNγ/IL2 FluoroSpot assay for clinical trial monitoring
Source: J Transl Med. 2016 Jun 14;14:175. doi: 10.1186/s12967-016-0932-7 (PMC4906590; doi:10.1186/s12967-016-0932-7)
Supplement: Supplementary file 1 — 10.1186/s12967-016-0932-7 Magnitude of background activity measured in unstimulated control wells. [file 12967_2016_932_MOESM1_ESM.pdf]

Additional file 1: Table S1: Magnitude of background activity measured in unstimulated control wells[a]

	IFNγ		IL2		IFNγ+IL2	
**Donor**	**Mean**	**SD**	**Mean**	**SD**	**Mean**	**SD**
S01	2	1.73	5	2.57	2	0.00
S02	3	4.93	4	4.49	1	0.64
S03	1	0.58	6	2.31	0	0.64
S04	0	0.58	5	2.80	1	0.58
S05	1	0.58	3	2.57	0	0.00
S06	1	1.13	7	3.21	0	0.00
S07	1	0.58	2	2.36	1	0.58
S08	2	1.30	10	3.72	0	0.46
S09	1	1.10	6	2.51	1	0.73
S10	2	1.59	3	1.55	0	0.25
S11	3	1.66	10	2.22	1	0.78
S12	1	1.23	5	2.11	0	0.70
S13	2	1.18	5	2.09	0	0.25
S14	4	2.56	3	3.39	1	1.08
S15	3	1.05	7	2.25	0	0.27
S16	2	1.21	8	3.02	1	0.79
S17	1	0.60	2	1.47	0	0.27
S18	2	2.14	9	2.95	1	1.06
S19	1	0.95	7	2.32	0	0.53
S20	2	1.58	9	2.78	0	0.51
S21	4	2.85	10	3.21	2	1.31
S22	1	0.88	7	3.16	0	0.17
S23	1	1.23	5	2.57	1	0.58
S24	1	0.64	1	0.43	0	0.18
S25	1	0.89	3	3.78	0	0.19
S26	1	1.18	3	2.04	0	0.19
S27	2	0.95	8	1.11	2	2.22
S28	3	2.52	3	3.57	1	0.58
S29	1	1.00	2	1.53	0	0.00
S30	1	0.58	1	1.92	1	0.58
**Mean**	**2**	**1.37**	**5**	**2.53**	**1**	**0.54**

[a]Mean values are the mean number of detected IFNγ, IL2 and IFNγ+IL2 SFC/2x10^5 PBMC in unstimulated control wells were media was added instead of any stimulatory agent (in triplicates; mean numbers of SFC of all performed FluoroSpot assays of each donor); SD = standard deviation
