# Supplementary material for: Validation of an IFNγ/IL2 FluoroSpot assay for clinical trial monitoring
Source: J Transl Med. 2016 Jun 14;14:175. doi: 10.1186/s12967-016-0932-7 (PMC4906590; doi:10.1186/s12967-016-0932-7)
Supplement: Supplementary file 2 — 10.1186/s12967-016-0932-7 Intra-assay variability of antigen-specific IFNγ, IL2, and IFNγ + IL2 responses ranging between 1-10 SFC/well. [file 12967_2016_932_MOESM2_ESM.pdf]

Additional file 2: Table S2A: Intra-assay variability of antigen-specific IFNγ responses ranging between 1-10 SFC/well[a]

Donor	Antigen	Replicate 1	Replicate 2	Replicate 3	Mean	SD	%CV
S12	BZLF1	2	12	8	7	5.03	68.63
S23	BZLF1	11	6	3	7	4.04	60.62
S28	BZLF1	8	9	5	7	2.08	28.39
S11	EBNA3A	4	3	2	3	1.00	33.33
S15	EBNA3A	4	8	7	6	2.08	32.87
S24	EBNA3A	3	8	4	5	2.65	52.92
S30	EBNA3A	10	8	4	7	3.06	41.66
**Mean**						**2.85**	**46.28**

Additional file 2: Table S2B: Intra-assay variability of antigen-specific IL2 responses ranging between 1-10 SFC/well[a]

Donor	Antigen	Replicate 1	Replicate 2	Replicate 3	Mean	SD	%CV
S17	EBNA3A	2	9	6	6	3.51	61.97
S24	EBNA3A	10	6	9	8	2.08	24.98
**Mean**						**2.80**	**43.48**

Additional file 2: Table S2C: Intra-assay variability of antigen-specific IFNγ+IL2 responses ranging between 1-10 SFC/well[a]

Donor	Antigen	Replicate 1	Replicate 2	Replicate 3	Mean	SD	%CV
S12	BZLF1	2	1	6	3	2.65	88.19
S13	BZLF1	2	7	5	5	2.52	53.93
S24	BZLF1	1	7	1	3	3.46	115.47
S11	EBNA3A	7	2	3	4	2.65	66.14
S14	EBNA3A	7	7	3	6	2.31	40.75
S15	EBNA3A	1	5	3	3	2.00	66.67
S24	EBNA3A	7	2	4	4	2.52	58.08
**Mean**						**2.59**	**69.74**

[a] Values represent the number of detected antigen-specific IFNγ (table S2A), IL2 (table S2B), and IFNγ+IL2 (table S2C) SFC/2x10^5 PBMC (stimulated with 1µg/ml BZLF1 or EBNA3A peptide pools) (data is not background subtracted) in the IFNγ/IL2 FluoroSpot assay; SD = standard deviation. CV = coefficient of variation.
