# Supplementary material for: Validation of an IFNγ/IL2 FluoroSpot assay for clinical trial monitoring
Source: J Transl Med. 2016 Jun 14;14:175. doi: 10.1186/s12967-016-0932-7 (PMC4906590; doi:10.1186/s12967-016-0932-7)
Supplement: Supplementary file 3 — 10.1186/s12967-016-0932-7 Intra-assay variability of antigen-specific IFNγ, IL2, and IFNγ + IL2 responses ranging between 11-50 SFC/well. [file 12967_2016_932_MOESM3_ESM.pdf]

Additional file 3: Table S3A: Intra-assay variability of antigen-specific IFNγ responses ranging between 11-50 SFC/well[a]

Donor	Antigen	Replicate 1	Replicate 2	Replicate 3	Replicate 4	Replicate 5	Mean	SD	%CV
S10	BZLF1	16	19	13	ND	ND	16	3.00	18.75
S17	BZLF1	22	29	28	ND	ND	26	3.79	14.38
S24	BZLF1	11	12	11	ND	ND	11	0.58	5.09
S01	EBNA3A	30	26	31	ND	ND	29	2.65	9.12
S10	EBNA3A	28	31	31	ND	ND	30	1.73	5.77
S12	EBNA3A	38	43	46	ND	ND	42	4.04	9.55
S19	PHA-L	35	35	45	44	38	39	4.83	12.25
**Mean**								**2.94**	**10.70**

Additional file 3: Table S3B: Intra-assay variability of antigen-specific IL2 responses ranging between 11-50 SFC/well[a]

Donor	Antigen	Replicate 1	Replicate 2	Replicate 3	Replicate 4	Replicate 5	Mean	SD	%CV
S09	BZLF1	39	39	43	ND	ND	40	2.31	5.73
S11	BZLF1	28	27	30	ND	ND	28	1.53	5.39
S14	BZLF1	37	31	34	ND	ND	34	3.00	8.82
S16	BZLF1	39	39	43	ND	ND	40	2.31	5.73
S18	BZLF1	32	37	37	ND	ND	35	2.89	8.17
S01	EBNA3A	33	29	21	ND	ND	28	6.11	22.08
S10	EBNA3A	26	26	20	ND	ND	24	3.46	14.43
S12	EBNA3A	47	44	51	ND	ND	47	3.51	7.42
S21	EBNA3A	42	38	38	ND	ND	39	2.31	5.87
**Mean**								**3.05**	**9.29**

Additional file 3: Table S3C: Intra-assay variability of antigen-specific IFNγ+IL2 responses ranging between 11-50 SFC/well[a]

Donor	Antigen	Replicate 1	Replicate 2	Replicate 3	Replicate 4	Replicate 5	Mean	SD	%CV
S11	BZLF1	19	18	21	ND	ND	19	1.53	7.90
S14	BZLF1	24	18	18	ND	ND	20	3.46	17.32
S16	BZLF1	26	23	26	ND	ND	25	1.73	6.93
S18	BZLF1	23	29	29	ND	ND	27	3.46	12.83
S01	EBNA3A	18	11	11	ND	ND	13	4.04	30.31
S10	EBNA3A	13	18	11	ND	ND	14	3.61	25.75
S12	EBNA3A	23	25	27	ND	ND	25	2.00	8.00
S20	EBNA3A	10	14	16	ND	ND	13	3.06	22.91
S21	EBNA3A	22	17	21	ND	ND	20	2.65	13.23
**Mean**								**1.70**	**16.13**

[a] Values represent the number of detected antigen-specific IFNγ (table 1A), IL2 (table 1B) and IFNγ+IL2 (table 1C) SFC/$2x10^5$ PBMC (stimulated with 1µg/ml BZLF1 or EBNA3A peptide pools) or mitogen-specific IFNγ (table 1A), IL2 (table 1B), and IFNγ+IL2 (table 1C) SFC /$5x10^4$ PBMC (stimulated with 2µg/ml PHA-L) (data is not background subtracted) in the IFNγ/IL2 FluoroSpot assay; ND = not done. SD = standard deviation. CV = coefficient of variation.
