# Supplementary material for: Validation of an IFNγ/IL2 FluoroSpot assay for clinical trial monitoring
Source: J Transl Med. 2016 Jun 14;14:175. doi: 10.1186/s12967-016-0932-7 (PMC4906590; doi:10.1186/s12967-016-0932-7)
Supplement: Supplementary file 4 — 10.1186/s12967-016-0932-7 Diagnostic specificity and sensitivity of the EBV-specific IFNγ/IL2 FluoroSpot. [file 12967_2016_932_MOESM4_ESM.pdf]

**Additional file 4: Table S4: Diagnostic specificity and sensitivity of the EBV-specific IFNγ/IL2 FluoroSpot[a]**

| Donor | EBV-serostatus | Antigen | Mean | SD | Antigen | Mean | SD |
|---|---|---|---|---|---|---|---|
| S02 | negative | BZLF1 | 0 | - | EBNA3A | 0 | - |
| S03 | negative | BZLF1 | 0 | - | EBNA3A | 0 | - |
| S04 | negative | BZLF1 | 0 | - | EBNA3A | 0 | - |
| S05 | negative | BZLF1 | 0 | - | EBNA3A | 0 | - |
| S06 | negative | BZLF1 | 0 | - | EBNA3A | 0 | - |
| S07 | negative | BZLF1 | 0 | - | EBNA3A | 0 | - |
| S01 | positive | BZLF1 | 117 | 4.51 | EBNA3A | 54 | 7.54 |
| S08 | positive | BZLF1 | 90 | 5.57 | EBNA3A | 12 | 1.53 |
| S09 | positive | BZLF1 | 274 | 16.27 | EBNA3A | 35 | 4.73 |
| S10 | positive | BZLF1 | 18 | 1.73 | EBNA3A | 42 | 3.06 |
| S11 | positive | BZLF1 | 411 | 10.31 | EBNA3A | 0 | - |
| S12 | positive | BZLF1 | 0 | - | EBNA3A | 102 | 2.59 |
| S13 | positive | BZLF1 | 0 | - | EBNA3A | 64 | 1.41 |
| S14 | positive | BZLF1 | 366 | 11.92 | EBNA3A | 0 | - |
| S15 | positive | BZLF1 | 84 | 4.36 | EBNA3A | 0 | - |
| S16 | positive | BZLF1 | 276 | 2.31 | EBNA3A | 29 | 4.93 |
| S17 | positive | BZLF1 | 26 | 3.79 | EBNA3A | 0 | - |
| S18 | positive | BZLF1 | 194 | 12.57 | EBNA3A | 16 | 2.52 |
| S19 | positive | BZLF1 | 67 | 6.81 | EBNA3A | 118 | 4.04 |
| S20 | positive | BZLF1 | 60 | 4.58 | EBNA3A | 65 | 8.48 |
| S21 | positive | BZLF1 | 0 | - | EBNA3A | 167 | 7.02 |
| S24 | positive | BZLF1 | 11 | 4.04 | EBNA3A | 0 | - |
| **Median** | | | **90** | | | **54** | |

[a] Illustrated is the total response (sum of IFNγ, IL2, and IFNγ+IL2 SFC/2x$10^5$ PBMC) of EBV-seropositive donors detected during the validation experiments (stimulated with 1µg/ml BZLF1 or EBNA3A peptide pools). Mean values are the mean number of antigen-specific total responses of each donor of all performed IFNγ/IL2 FluoroSpot assays; SD = standard deviation.
